# Intramuscular Administration of Drotaverine Hydrochloride Decreases Both Incidence of Urinary Retention and Time to Micturition in Orthopedic Patients under Spinal Anesthesia: A Single Blinded Randomized Study

**DOI:** 10.1155/2015/926953

**Published:** 2015-06-21

**Authors:** Dariusz Tomaszewski, Mariusz Bałkota

**Affiliations:** Department of Anesthesiology and Intensive Therapy, Military Institute of Medicine, 128 Szaserów Street, 04-141 Warsaw, Poland

## Abstract

*Purpose*. Postoperative urinary retention (POUR) increases the duration of hospitalization and frequency and risk of urinary bladder catheterization. The objective of this study was to analyze the efficacy of intramuscularly administered drotaverine hydrochloride in the prevention of POUR in orthopedic patients. *Methods*. Two hundred and thirty patients 17–40 years of age undergoing lower limb orthopedic procedures under spinal anesthesia were enrolled in the study. The study group received 40 mg of drotaverine hydrochloride intramuscularly; the second group was the control. The main outcome measure was (1) the time to micturition and (2) the incidence of urinary bladder catheterization and time to catheterization. *Results*. Two hundred and one patients of 230 enrolled participants completed the study. Compared to the control group, the male patients in study group exhibited a shorter time to spontaneous micturition (441 versus 563 minutes, 95% CI of the difference of means between 39 and 205 minutes) and a lower incidence of urinary bladder catheterization (4/75 versus 10/54) (RR 0.29, 95% CI: 0.1–0.87; *P* = 0.0175). *Conclusions*. Intramuscular administration of drotaverine hydrochloride decreased the time to spontaneous micturition and decreased the incidence of urinary bladder catheterization in male patients who underwent orthopedic surgery under spinal anesthesia. This trial is registered with NCT02026427.

## 1. Introduction

The incidence of postoperative urinary retention (POUR) in surgical patients varies from 5% to 70% [1]. Its etiology is complex; however, male gender, old age, anesthesia duration longer than 200 minutes, and type of surgery have been identified as risk factors. Spinal anesthesia itself may influence urinary bladder function. The duration of those disturbances depends on both the potency and administered dose of local anesthetics [2].

POUR can be diagnosed clinically (discomfort and pain in the lower part of the abdomen) and by ultrasound imaging when the volume of urine inside the urinary bladder exceeds 400 mL [3] to 600 mL [4] and when the inability to void lasts longer than 30 minutes [4].

POUR increases the duration of a patient's stay in the postoperative ward [5] and the incidence of urinary bladder catheterization [2]. As with all invasive medical procedures, urinary bladder catheterization is associated with an increased incidence of complications. The most important complications are trauma, the formation of abscesses and urinary fistulae, symptomatic bacterial infections, and urosepsis [6].

Urinary bladder function may be influenced by different medications, such as anticholinergics, beta-adrenolytics, and sympathomimetics. Muscarinic drugs increase intravesical pressure, leading to hyperactive detrusor contraction. Anticholinergics decrease detrusor contraction, and decreased tension of the urinary bladder may lead to urinary retention. Alpha 2 agonists and antagonists influence urinary bladder function via the alpha-receptors in smooth muscle cells located in the upper and lower urinary tract [1].

Bałkota et al. [7] analyzed the influence of neostigmine hydrochloride administration on the incidence of POUR in orthopedic patients under spinal anesthesia; however, the results were not satisfactory. They observed a higher incidence of urinary retention in the study group compared with the control one, probably as an effect of increased tone of smooth muscles of urinary bladder. Those findings led us to undertake the current project.

Drotaverine hydrochloride (ATC code: A03AD02) is an antispasmodic drug structurally related to papaverine. It is a selective inhibitor of phosphodiesterase 4, with no anticholinergic effects (http://www.drugbank.ca/drugs/DB01400/; access: 14.07.2014 09:10). Drotaverine hydrochloride was approved by the Polish Office for Registration of Medicinal Products, Medical Devices and Biocidal Products (permit number 0307). The indications to drotaverine administration include contractions of the biliary ducts, urinary tract, and digestive smooth muscles; also dysmenorrhea and imminent abortion.

Thus, the objective of the current study was to analyze the influence of intramuscularly administered drotaverine hydrochloride, in the prevention of POUR in orthopedic surgery under spinal anesthesia.

Our hypothesis was that administration of drotaverine hydrochloride, a spasmolytic agent, reduces the incidence of urinary retention in patients under spinal anesthesia.

## 2. Methods

The study was planned as a randomized controlled trial. After the commencement of the study its design was not modified.

We received local ethical committee approval (40/WIM/2010), and informed consent was obtained from all of the subjects. The participants, aged 17–40 years, were hospitalized at the Department of Orthopedics and Department of Traumatology of Military Institute of Medicine, Warsaw, Poland. The inclusion criteria were (1) being under 40 years of age, (2) no history of urologic problems, and (3) surgery under spinal anesthesia. Patients in whom (1) opioids were administered during the surgery, (2) general anesthesia was necessary, or (3) the urinary bladder catheterization was performed during the surgery were excluded from the study.

The patients included in the study were assigned randomly to the study and control groups by the researchers. The simple randomization method (flipping a coin) was used. The patients were not informed to which group, control or study, they were assigned. The participants were not premedicated orally. All of the patients were asked to empty their urinary bladders prior to arriving at the operating theater. When standard monitoring (continuous electrocardiogram, noninvasive blood pressure, and pulse oximetry) was started, an intravenous premedication with midazolam was administered, followed by lumbar spinal anesthesia with hyperbaric 0.5% solution of bupivacaine hydrochloride. When the proper level of regional anesthesia was established, 40 mg of drotaverine hydrochloride was administered intramuscularly to the patients in the study group. A nurse anesthetist made this injection. In this time patients were already separated from the operation site by the curtain. Neither drotaverine hydrochloride nor placebo was administered to the control group. The crystalloid infusion during surgery was left to the discretion of anesthetists and not* a priori* defined. During the surgery, an adequate level of sedation, but not deeper than second grade according to the Ramsay scale, was achieved with midazolam and/or propofol.

All of the participants received a questionnaire in which we asked the following questions: (1) the time of micturition; (2) the time at which discomfort/pain appeared in the lower abdomen, if at all; (3) the time at which the catheter was inserted into the urinary bladder; (4) the time of micturition following removal of the catheter from the bladder; (5) the time at which the signs of regional anesthesia disappeared and the patient noted that there were no altered sensations within the lower part of the body, including sacral dermatomes. The questionnaires were collected the day following surgery by the investigator who did not know to which group, study or control, patient was allocated.

Our primary outcome measure was

1. the time to micturition, defined as the time (in minutes) from the administration of the local anesthetic to urination,
2. the incidence of urinary bladder catheterization and time to catheterization, defined as the time (in minutes) from the onset of discomfort or pain in the lower abdomen until the catheterization was performed.

We also analyzed (3) the time at which discomfort or pain appeared in the lower abdomen and the duration (in minutes) of this discomfort or pain; we assumed that those sensations corresponded with urinary retention.

## 3. Statistical Analysis

### 3.1. Sample Size Calculation

According to Kreutziger et al. [8], the incidence of urinary bladder catheterization between 16 and 40 years of age was 52.9% in female and 6.3% in male patients. Assuming the lower of these values, the sample size for 95% confidence level is 87 patients. In our study we added additional 15% to this value.

The data were analyzed using R statistical software, version 3.1.0 (R: a language and environment for statistical computing (2008), R Development Core Team, R Foundation for Statistical Computing, Vienna, Austria; http://www.R-project.org/). Demographic information (age, gender, body mass, and height), administered dose of local anesthetic, level of regional anesthesia, volume of crystalloids administered intra- and postoperatively, duration of anesthesia, and parameters (1)–(4) mentioned above were analyzed with descriptive statistics.

The differences between the two groups in gender, incidence of discomfort or pain in the lower abdomen, and urinary bladder catheterization were analyzed with a chi square test. The analysis of the other data was begun by using the Shapiro-Wilk test to determine whether they were normally distributed. If the dataset was normally distributed, Levene's test was used to analyze the equality of variances. If the variances were equal, the differences between the two groups were analyzed with Student's *t*-test. If the variances were not equal, the Mann-Whitney *U* test was used. In the statistical analysis, *P* < 0.05 was considered statistically significant.

## 4. Results

**Figure 1:**
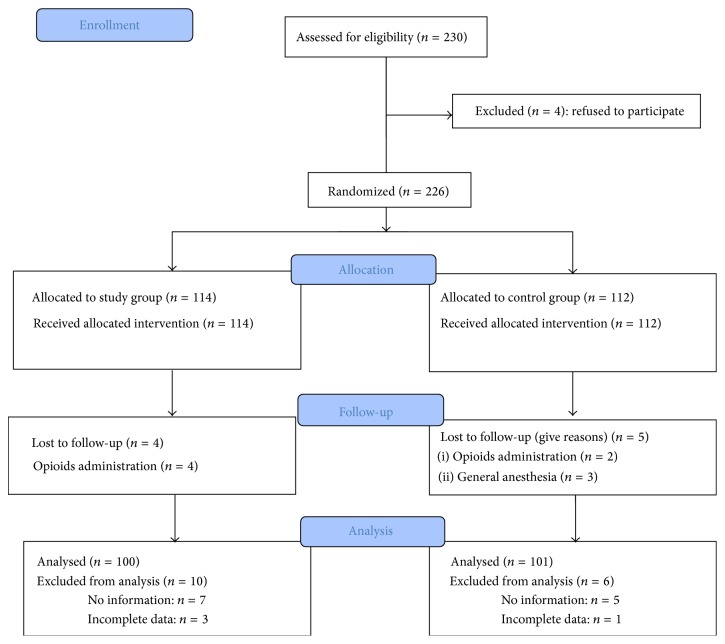
Flow of the participants in the study.

Data were collected between October 2010 and March 2014. The study was terminated after completing all the data. Of 230 patients enrolled in the study, 201 patients were analyzed; the patient flow is shown in Figure 1. The demographic data of the participants are presented in Table 1, and the types of surgery are presented in Table 2.

The study group included 75 men and 25 women, and the control group included 54 men and 47 women; this difference was statistically significant (*P* = 0.002). There was no significant difference in age between the groups (*P* = 0.123). The body mass and height of the patients in the study group were higher compared with the control group (*P* = 0.005 and 0.044, resp.).

The levels of anesthesia of the two groups were similar (*P* = 0.239); however, the administered dose of local anesthetic was higher in the study group (*P* = 0.001). There were no differences in volume of crystalloids administered intra- and postoperatively (*P* = 0.472 and 0.799, resp.). The mean duration of the surgery was similar in both groups (*P* = 0.533).

In the study group, 26/100 (26.0%) of the patients had discomfort or pain in the lower abdomen, compared with 41/101 (40.6%) patients in the control group. This difference was statistically significant (*P* = 0.041). Urinary bladder catheterization was necessary in 6/100 (6.0%) patients in the study group, compared with 18/101 (17.8%) of patients in the control group; the difference was statistically significant (RR 0.337, 95% CI: 0.139–0.813; *P* = 0.018). There were no differences between the groups in time from the onset of regional anesthesia to the beginning of discomfort or pain in the lower abdomen (*P* = 0.907); duration of discomfort or pain (*P* = 0.114); or time from the onset of anesthesia to urinary bladder catheterization (*P* = 0.382). Our data, only partially completed, on the volume of excreted urine showed that, despite clinically observed differences between the groups (median volume of urine of 500 and 700 mL in the study and control groups, resp.), the difference was not statistically significant (*P* = 0.204).

We were not satisfied with the fact that the two groups were not demographically similar. Although it is quite difficult to discuss with the flipping coin, we performed additional analysis and compare either women in study and control group or men in study and control group. The results of this analysis are shown in Tables 3 and 4.

We found that amongst women in study group, in comparison to control one, there were no differences regarding age of the patients (*P* = 0.7761), body mass (*P* = 0.7581), and their height (*P* = 0.8916). The dose of local anesthetic, the level of anesthesia, the volume of crystalloids transfused intra- and postoperatively, and mean duration of surgery were also similar (*P* values of 0.2611, 0.1517, 0.9317, 0.5663, and 0.6522, resp.). Thirty-six percent (9/25) of women in study and 46.8% (22/47) in control group felt discomfort in the lower part of the abdomen; the difference was not significant (*P* = 0.1179). There were no differences in the onset time of the discomfort between women in study and control group (*P* = 0.8447) and its duration (*P* = 0.1598). In 8% (2/25) of women in study and in 17% (8/47) in control group, catheterization of the urinary bladder was necessary; the difference was not significant (RR 0.47, 95% CI: 0.108–2.047; *P* = 0.292). The time from onset of anesthesia to bladder catheterization was similar in both groups (*P* = 0.8948). The time to spontaneous micturition was also similar (*P* = 0.9906). There were no differences in the urine volume (583 mL in study versus 662 mL in control group; *P* = 0.8571).

There were no differences in age of the male patients in study and control group (*P* = 0.7144), their body mass (*P* = 0.6809), and height (*P* = 0.5928). The dose of local anesthetic, the level of anesthesia, the volume of crystalloids transfused intra- and postoperatively, and mean duration of surgery were also similar (*P* = 0.232, 0.5447, 0.3537, 0.2655, and 0.286, resp.). Nineteen of seventy-five patients (22.7%) in study and 19/54 (35.2%) in control group felt discomfort in the lower part of the abdomen; the difference was not significant (*P* = 0.1179). There were no differences in the onset time of the discomfort between men in study and control group (*P* = 0.7155) and its duration (*P* = 0.7982). In 5.3% (4/75) of men in study and in 18.5% (10/54) in control group, catheterization of the urinary bladder was necessary; this difference was statistically significant (RR 0.288, 95% CI: 0.095–0.87; *P* = 0.0175). The time from onset of anesthesia to bladder catheterization was similar in both groups (*P* = 0.1772). In male patients in study group the mean time to spontaneous micturition was shorter in study group, in comparison to control one (*P* = 0.0067). We found no differences in the urine volume (618 mL in study versus 919 mL in control group; *P* = 0.1876).

**Figure 2:**
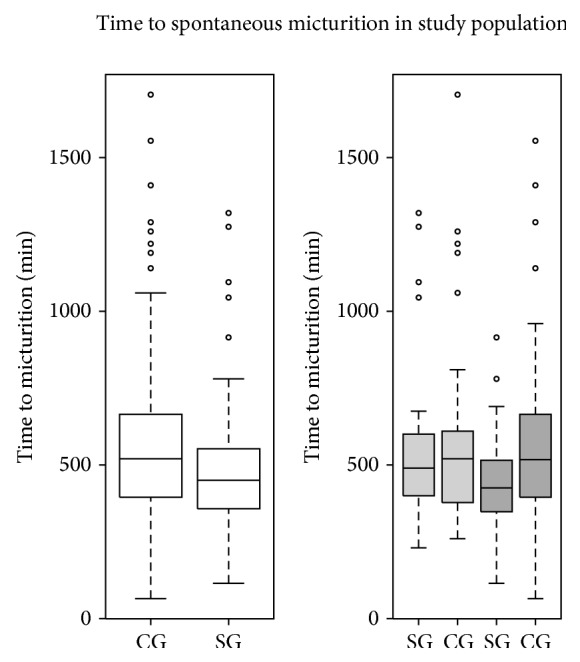
The comparison of the time (in minutes) from the onset of spinal anesthesia to spontaneous micturition in study population. White boxplot: both men and women; light grey: women; dark grey: men.

The time to spontaneous micturition in study population was shown in Figure 2.

No important injuries or unintended effects of the intramuscular administration of drotaverine hydrochloride were observed.

## 5. Discussion

Spinal anesthesia is frequently administered in patients undergoing orthopedic surgery. The benefits of regional procedures are well known. However, there are some risk factors of urinary retention after spinal anesthesia. Spinal anesthesia may influence urinary bladder functions, leading to urinary retention. The intrathecal administration of long-acting local anesthetics and opioids was related to higher incidence of POUR [1]. Also, bilateral spinal anesthesia was related to a higher incidence of disturbances in micturition and time to first voiding, in comparison with unilateral procedures [9]. In their analysis, Keita et al. [4] found that predictive factors for postoperative urinary retention were age ≥ 50 years, duration of surgery ≥ 60 minutes, duration of anesthesia ≥ 80 minutes, quantity of intraoperative fluids ≥ 750 mL, and bladder volume on entry in the postanesthesia care unit ≥ 270 mL. The incidence of POUR after orthopedic surgery was more frequent in patients with a previous history of hypertension and diabetes mellitus [10].

Postoperative urinary retention and its possible relevance to spinal anesthesia can reduce the use of this procedure in orthopedic patients, especially in one-day surgeries, where the spontaneous voiding after regional anesthesia is one of the patient's hospital discharge criteria.

In this study, we found that intramuscular administration of drotaverine hydrochloride significantly decreased both the time from spinal anesthesia to spontaneous micturition and the frequency of urinary bladder catheterization.

Despite the interesting results, our study has important limitations.

The study was only single blinded. The patients were not informed to which group, control or study, they were assigned. In the study group drotaverine was administered after the proper level of regional anesthesia was obtained and after intravenous administration of midazolam. Furthermore, the patients were separated from the operation site by the curtain. Thus the participants did not know their allocation.

The doses of local anesthetic to achieve the proper level of regional anesthesia were administered according to the patients' height. The volumes of crystalloids administered intra- and postoperatively were estimated in compliance with the patients' basal need and the duration of preoperative fasting.

The diagnosis of POUR in our study was only made clinically. We assumed that a sensation of discomfort or pain in the lower part of the abdomen was equivalent to an ultrasound evaluation. In the literature, POUR was diagnosed when patients were unable to void and the volume of urine in the bladder exceeded 400–600 mL [11]. The incidences of discomfort or pain in the lower part of the abdomen in our study were similar to those observed by Feliciano et al. [5]. A strength of our study is that the participants underwent orthopedic surgeries, thereby excluding the possibility that discomfort or pain had its origin in surgery.

However, the routine ultrasound examination of bladder volume was not carried out. In some patients who felt the discomfort in the lower abdomen the duration of this sensation was relatively long. Some of the participants wanted to void spontaneously, thus refusing the catheterization of the urinary bladder.

There were significantly more men than women in the study group compared with the control group. Because the authors of this study were not satisfied with the gender differences between the study and control group, an additional analysis was performed. The efficacy of drotaverine hydrochloride was observed in male patients, but not in women. POUR is more frequent in men [1, 12], although the results of Kreutziger et al. [8] and Ehrenberg et al. [9] were different. The correlation between the dose and potency of local anesthetic administered into the cerebrospinal fluid and the incidence of POUR has also been described [2].

It was shown that detrusor contraction is abolished within a few minutes (2 to 5 minutes) after intrathecal administration of local anesthetic, and muscle contraction recovery depends on the duration of the sensory block above the 3rd and 4th sacral segments [1]. Spinal anesthesia does not influence the functions of the bladder sphincter muscle. So, the sphincter of the urinary bladder could be a point of antispasmodic action of drotaverine. The mechanism of the efficacy of drotaverine hydrochloride could be its action on the smooth muscles of the urinary bladder.

Drotaverine hydrochloride, by its inhibitory effect on phosphodiesterase, increases the concentration of cyclic adenosine monophosphate (cAMP). Increased concentration of cAMP leads to activation of myosin light-chain kinase (MLCK). The phosphorylation of MLCK decreases its affinity to calcium-calmodulin complex; the inactive form of MLCK leads to muscle relaxation. Drotaverine hydrochloride in vitro inhibits phosphodiesterase (PDE) IV, but not PDE III and V [13].

Following parenteral administration, drotaverine is quickly and completely absorbed [13]. After intravenous administration, its elimination half-time was 9.33 ± 1.02 hours, plasma clearance was 243 ± 51 mL/min, and the volume of distribution was 195 ± 48 liters [14]. Drotaverine is metabolized in the liver [13, 14]. More than 50% of the administered dose of drotaverine is excreted with urine and about 30% with stool [13].

To our knowledge, the efficacy of drotaverine hydrochloride in the prevention of urinary retention after spinal anesthesia has not previously been studied. An analysis of the Medline database indicated that there were 143 records on “drotaverine,” the majority of which applied to obstetric cases. We found no studies on the efficacy of drotaverine hydrochloride in POUR.

In our study, we observed no unintended effects of administration of drotaverine. Generally, this drug is considered safe; however, it is contraindicated in patients with severe renal, liver, and heart failure and also in children. Nausea, headaches, dizziness, arrhythmias, hypotension, and allergic reactions were also noted [13].

We realize that drotaverine hydrochloride was registered in limited number of countries. However, our results suggesting that intramuscular administration of spasmolytic agent decreases time to spontaneous micturition and incidence of urinary bladder catheterization in male orthopedic patients under spinal anesthesia may be important in future studies on postoperative urinary retention.

## 6. Conclusions

Intramuscular administration of drotaverine hydrochloride decreased the time to spontaneous micturition and decreased the incidence of urinary bladder catheterization in male patients who underwent orthopedic surgery under spinal anesthesia.

## Figures and Tables

**Table 1:** Summary of analyzed data of the study group (group I) and control group (group II).

Variable	Study group (I)	Control group (II)
Age, in years^*∗∗*^	29 (28–31)	28 (26–29)
Height, in cm^*∗∗*^	178 (176–180)	176 (173–178)
Body mass, in kg^*∗*^	83 (80–86)	76 (73–80)
Dose of local anesthetic, in mg^*∗∗*^	17.5 (17.5-17.5)	17.5 (17.0–17.5)
Level of anesthesia, thoracic dermatome^*∗∗*^	10 (10-10)	10 (10-10)
Duration of anesthesia, in minutes^*∗∗*^	365 (338–395)	375 (345–410)
Volume of crystalloids transfused during surgery, in mL^*∗∗*^	1500 (1500–2000)	1500 (1500–2000)
Volume of crystalloids transfused after surgery, in mL^*∗∗*^	1500 (1000–1500)	1500 (1000–1500)
Time to onset of discomfort/pain, in minutes^*∗∗*^	298 (223–338)	270 (230–330)
Duration of discomfort/pain, in minutes^*∗∗*^	60 (53–80)	90 (60–90)
Time to micturition, in minutes^*∗∗*^	450 (420–472)	520 (412–473)
Time to urinary bladder catheterization, in minutes^*∗*^	320 (233–408)	281 (233–330)
Duration of surgery, in minutes^*∗∗*^	60 (55–65)	60 (55–65)

Data are presented as mean/95% confidence intervals for mean (data with normal distribution^*∗*^) or median/95% confidence intervals for median (data violating normality assumption^*∗∗*^).

**Table 2:** Types of surgical procedures in the study and control groups.

Type of surgery	Study group	Control group
Knee ligament reconstruction	39	25
Knee arthroplasty	38	46
Fixation of the fractures of the lower limbs	11	11
Chondroplasty	5	5
Hallux reconstruction	2	1
Achilles tendon repair	1	3
Cheilectomy	0	2
Implant removal	1	3
Surgical biopsy of tibial tumor	1	3
Snapping hip surgery	1	0
Arthrodesis of the ankle joint	1	0
Removal of foreign body from shin	0	1
Lateral retinacular release	0	1

**Table 3:** Summary of analyzed data of women in the study group (group I) and control group (group II).

Variable	Study group (I)	Control group (II)
Age, in years^*∗∗*^	26 (22–30)	25 (24–27)
Height, in cm^*∗∗*^	168 (165–173)	169 (165–170)
Body mass, in kg^*∗*^	63 (60–69)	63 (61–66)
Dose of local anesthetic, in mg^*∗∗*^	16.5 (16–17.5)	16 (16–16.5)
Level of anesthesia, thoracic dermatome^*∗∗*^	9 (8–10)	10 (10-10)
Duration of anesthesia, in minutes^*∗∗*^	365 (255–395)	360 (310–420)
Volume of crystalloids transfused during surgery, in mL^*∗∗*^	1500 (1500–2000)	1500 (1500–2000)
Volume of crystalloids transfused after surgery, in mL^*∗∗*^	1000 (1000–1500)	1000 (1000-1000)
Time to onset of discomfort/pain, in minutes^*∗∗*^	230 (188–300)	265 (185–300)
Duration of discomfort/pain, in minutes^*∗∗*^	60 (15–170)	90 (75–150)
Time to micturition, in minutes^*∗∗*^	490 (420–595)	520 (410–570)
Time to urinary bladder catheterization, in minutes^*∗*^	240 (constant data)	263 (209–317)
Duration of surgery, in minutes^*∗∗*^	65 (50–85)	55 (40–75)

Data are presented as mean/95% confidence intervals for mean (data with normal distribution^*∗*^) or median/95% confidence intervals for median (data violating normality assumption^*∗∗*^).

**Table 4:** Summary of analyzed data of men in the study group (group I) and control group (group II).

Variable	Study group (I)	Control group (II)
Age, in years^*∗∗*^	30 (29–32)	29 (28–32)
Height, in cm^*∗∗*^	180 (179-180)	180 (180–182)
Body mass, in kg^*∗*^	89 (86–91)	88 (84–91)
Dose of local anesthetic, in mg^*∗∗*^	17.5 (17.5-17.5)	17.5 (17.5-17.5)
Level of anesthesia, thoracic dermatome^*∗∗*^	10 (10-10)	10 (10-10)
Duration of anesthesia, in minutes^*∗∗*^	365 (335–405)	380 (348–413)
Volume of crystalloids transfused during surgery, in mL^*∗∗*^	1500 (1500–2000)	1500 (1500–2000)
Volume of crystalloids transfused after surgery, in mL^*∗∗*^	1500 (1500-1500)	1500 (1500-1500)
Time to onset of discomfort/pain, in minutes^*∗∗*^	320 (230–400)	310 (235–390)
Duration of discomfort/pain, in minutes^*∗∗*^	60 (55–70)	85 (30–90)
Time to micturition, in minutes^*∗∗*^	425 (395–460)	518 (438–563)
Time to urinary bladder catheterization, in minutes^*∗*^	361 (248–475)	296 (212–380)
Duration of surgery, in minutes^*∗∗*^	60 (55–65)	60 (55–70)

Data are presented as mean/95% confidence intervals for mean (data with normal distribution^*∗*^) or median/95% confidence intervals for median (data violating normality assumption^*∗∗*^).
